# Analgesic, Anti-Inflammatory, Cytotoxic Activity Screening and UPLC-PDA-ESI-MS Metabolites Determination of Bioactive Fractions of *Kleinia pendula*

**DOI:** 10.3390/molecules25020418

**Published:** 2020-01-20

**Authors:** Mohammad Alfaifi, Abdulrhman Alsayari, Narasimman Gurusamy, Justin Louis, Serag Eldin Elbehairi, Kumar Venkatesan, Sivakumar Annadurai, Yahya I. Asiri, Ali Shati, Kamel Saleh, Helmi Alboushnak, Heba Handoussa, Abdullatif Bin Muhsinah, Amira Abdel Motaal

**Affiliations:** 1 Department of Biology, Faculty of Science, King Khalid University, Abha 9004, Saudi Arabia; serag@kku.edu.sa (S.E.E.); aaalshati@kku.edu.sa (A.S.); kasaleh@kku.edu.sa (K.S.); halboushnak@kku.edu.sa (H.A.); 2 College of Pharmacy, King Khalid University, Abha 9004, Saudi Arabia; alsayari@kku.edu.sa (A.A.); gurunaras@gmail.com (N.G.); justin48@gmail.com (J.L.); kumarve@kku.edu.sa (K.V.); sannadurai@kku.edu.sa (S.A.); yialmuawad@kku.edu.sa (Y.I.A.); ajmohsnah@kku.edu.sa (A.B.M.); aabdulmtaal@kku.edu.sa (A.A.M.); 3 Department of Pharmaceutical Biology, Faculty of Pharmacy and Biotechnology, German University in Cairo, Cairo 11835, Egypt; heba.handoussa@guc.edu.eg

**Keywords:** *Kleinia pendula* (Forssk.) DC., fraction, metabolite, cytotoxic, analgesic, anti-inflammatory

## Abstract

*Kleinia pendula* (Forssk.) DC. is a prostrate or pendent dark green succulent herb found in the southwestern mountain regions of Saudi Arabia. The literature survey of the plant reveals a lack of phytochemical and pharmacological studies, although traditional uses have been noted. The objective of the present work was to assess the in vivo analgesic and anti-inflammatory activities, as well as, the in vitro cytotoxic potential of the fractions of *Kleinia pendula*, and correlate these activities to the plant metabolites. The methanolic extract of *Kleinia pendula* was subjected to fractionation with *n*-hexane, ethyl acetate, chloroform, *n*-butanol, and water. The fractions were screened for their analgesic and anti-inflammatory activities, as well as cytotoxic activity against breast, liver, and colon cancer cell lines. The *n*-hexane and chloroform fractions of *Kleinia pendula* showed significant cytotoxic activity against all three cancer cell lines tested. The ethyl acetate and chloroform fractions showed significant analgesic and anti-inflammatory activities. The metabolites in these three active fractions were determined using UPLC-PDA-ESI-MS. Thus, the analgesic and anti-inflammatory activities of the plant were attributed to its phenolic acids (caffeoylquinic acid derivatives, protocatechuic, and chlorogenic acids). While fatty acids and triterpenoids such as (tormentic acid) in the hexane fraction are responsible for the cytotoxic activity; thus, these fractions of *Kleinia pendula* may be a novel source for the development of new plant-based analgesic, anti-inflammatory, and anticancer drugs.

## 1. Introduction

**Figure 1:**
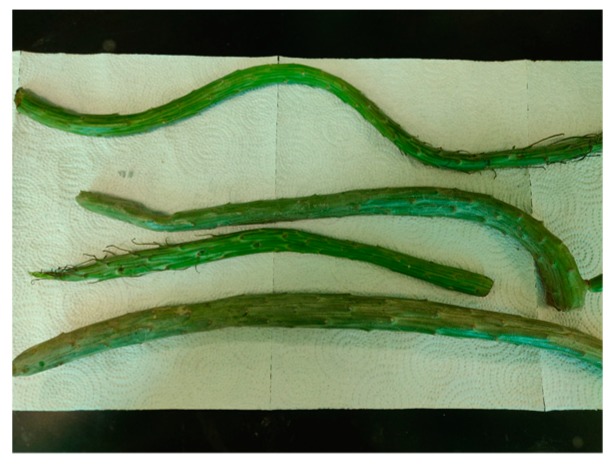
Kleinia pendula.

Historically, natural products derived from plants and animals have been the source of virtually all medicinal preparations. Natural products or direct derivatives of natural products have contributed to 49% of cancer medications approved by the U.S. Food and Drug Administration (FDA) between 1981 and 2014 [1]. Natural product collections exhibit certain advantages over their synthetic counterparts. They exhibit a high degree of stereochemistry and a wide range of pharmacophores. Since they are natural metabolites, natural products possess the unique property of metabolite-likeness. This property renders them as likely substrates of transporter systems, which ensure their delivery to intracellular sites of action [2]. The genus *Kleinia* is a flowering plant comprised of 40 species that are distributed throughout Somalia, the Middle East, Madagascar, and India [3,4]. Three species of this genus are commonly found in the southern regions of Saudi Arabia: *K. odora* (Forssk) DC, *K. deflersii* (O. Schwartz), and *K. pendula* (Forssk.) Sch. Bip [5,6,7]. Several species of *Kleinia* are known to be rich sources of oxygenated sesquiterpenoids, such as germacrane, oplopane abrotanifolon derivatives, and lupane-type tri-terpenoids [8,9,10,11]. The plant of interest, *Kleinia pendula* (Forssk.) DC., (Figure 1) has been used in Saudi and Yemeni traditional medicine to treat otitis (inflammation of the ear) [12]. The decoction of the fresh succulent *Kleinia pendula* has been used to treat swollen body parts in Ethiopian traditional medicine [13]. In the southern part of Somalia, the finely chopped aerial parts of the *Kleinia pendula* have been used as an insectifuge and/or insecticide in traditional and local veterinary practice.

The only report on phytochemical investigations on this plant pertains to the isolation and characterization of biologically active sesquiterpene alcohol, 4αH-eudesm-5α-ol, from the steam volatile oil of the aerial parts of *Kleinia pendula* [14]. The in vitro anticancer, antimicrobial, antioxidant, antiplasmoidal, antileishmanial, and antitrypanosomal activities of the methanol and aqueous extracts of selected medicinal plants, including *Kleinia pendula*, have been reported [3,12]. The lack of extensive phytochemical and pharmacological studies on this plant prompted the present study. In this work, the analgesic and anti-inflammatory potential of the fractions of *Kleinia pendula* on animal models have been demonstrated for the first time. Additionally, cytotoxic activity screening of the fractions against three human cancer cell lines has been reported. The phytochemical characterization of the bioactive fractions was carried out using an Ultra Performance Liquid Chromatography-Photodiode Array-Electrospray Ionization-Mass spectrometry (UPLC-PDA-ESI-MS) method.

## 2. Results

### 2.1. Acute Oral Toxicity Studies

The n-hexane, chloroform and ethyl acetate fractions of *Kleinia pendula* were subjected to an acute oral toxicity study at the dose of 2000 mg/kg body weight in female mice, as per the Organization for Economic Co-operation and Development (OECD) 420 guidelines. Gross behavioral parameters were closely observed for the first 3 h then at an interval of every 4 h during the next 48 h, such as mortality, diarrhea, respiratory problems, Straub tail, piloerection, convulsions were carefully examined in each animal. Treatment with the fractions of *Kleinia pendula* did not produce any mortality at a dose of 2000 mg/kg (p.o.), and there were no changes in the gross behavior of the animals. Moreover, the animals were kept under observation for two weeks after testing. Food and water intake had no significant difference among the groups studied.

### 2.2. Analgesic Activity of Fractions of Kleinia pendula

**Figure 2:**
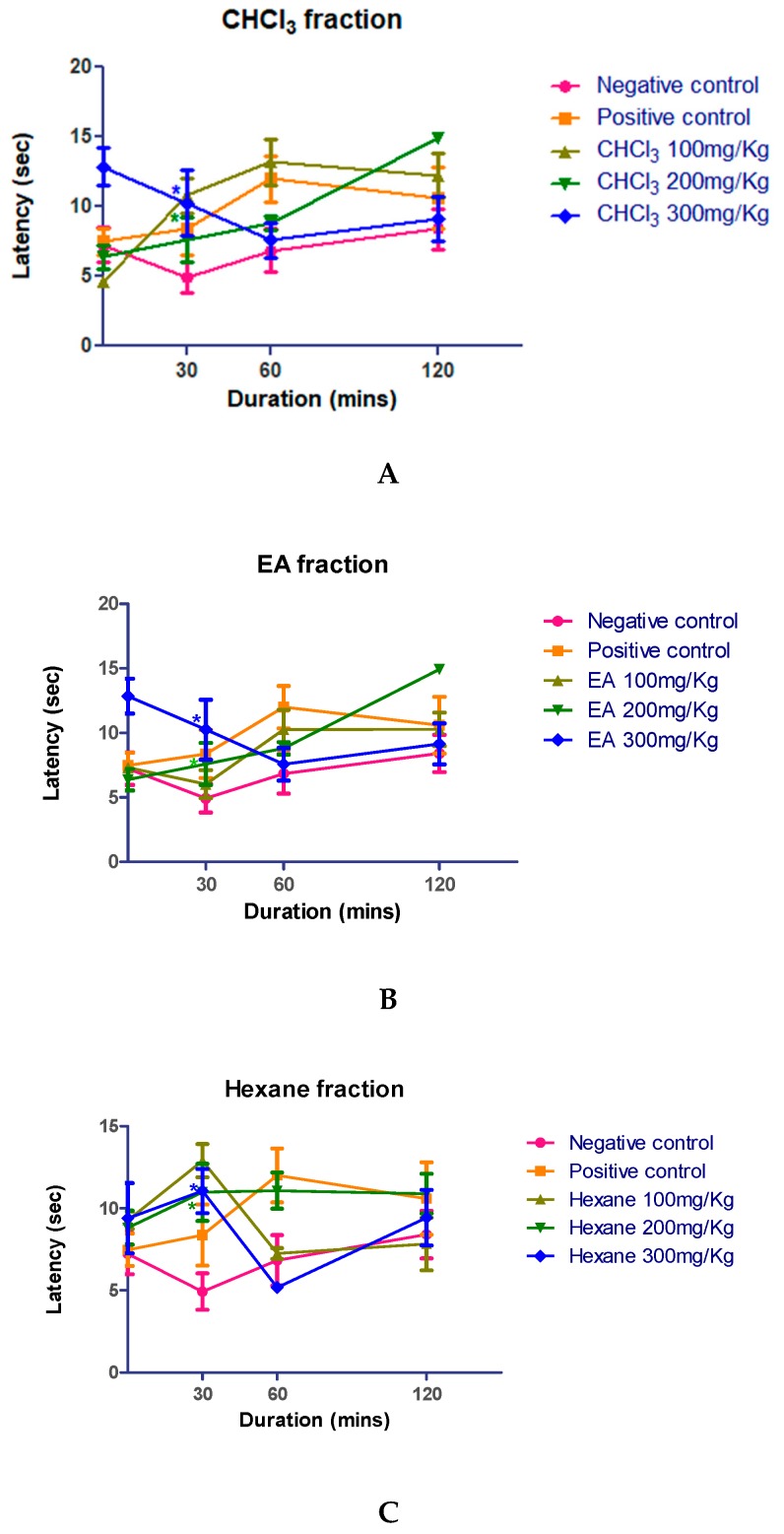
Analgesic activity of various fractions of *Kleinia pendula* at different doses and time points. The (CHCl_3_) fraction at doses of 100, 200, and 300 mg/kg body weight of the animal (**A**) exhibited analgesic activity. Ethyl acetate (EA) fraction at doses of 100, 200, and 300 mg/kg body weight of the animal (**B**) exhibited analgesic activity. The hexane fraction at doses of 100, 200, and 300 mg/kg body weight of the animal (**C**) exhibited low analgesic activity. Diclofenac sodium (10 mg/kg) was used as a positive control, and standard saline was used as negative control. The values are represented as mean ± SEM. Statistical analysis was done using one-way analysis of variance using the Dunnett’s posthoc test.

Chloroform, ethyl acetate, and *n*-hexane fractions were individually screened for analgesic activity. The doses were determined based on acute oral toxicity studies. The mice were administered with 100, 200, and 300 mg/kg fractions of *Kleinia pendula* followed by the measurement of analgesic activities using Eddy’s hot plate method at 0, 30, 60, and 120 min after the drug administration. As shown in Figure 2, the chloroform, ethyl acetate, and *n*-hexane fractions of *Kleinia pendula* exhibited a significant increase in analgesic activity at 30 min, comparable to the effect of the standard drug diclofenac sodium (10 mg/kg).

### 2.3. Anti-Inflammatory Activity of Fractions of Kleinia Pendula

The animals treated with standard saline showed a progressively increased inflammation at 60, 120, and 180 min. The chloroform and ethyl acetate fractions of *Kleinia pendula* were screened for anti-inflammatory activity.

**Figure 3:**
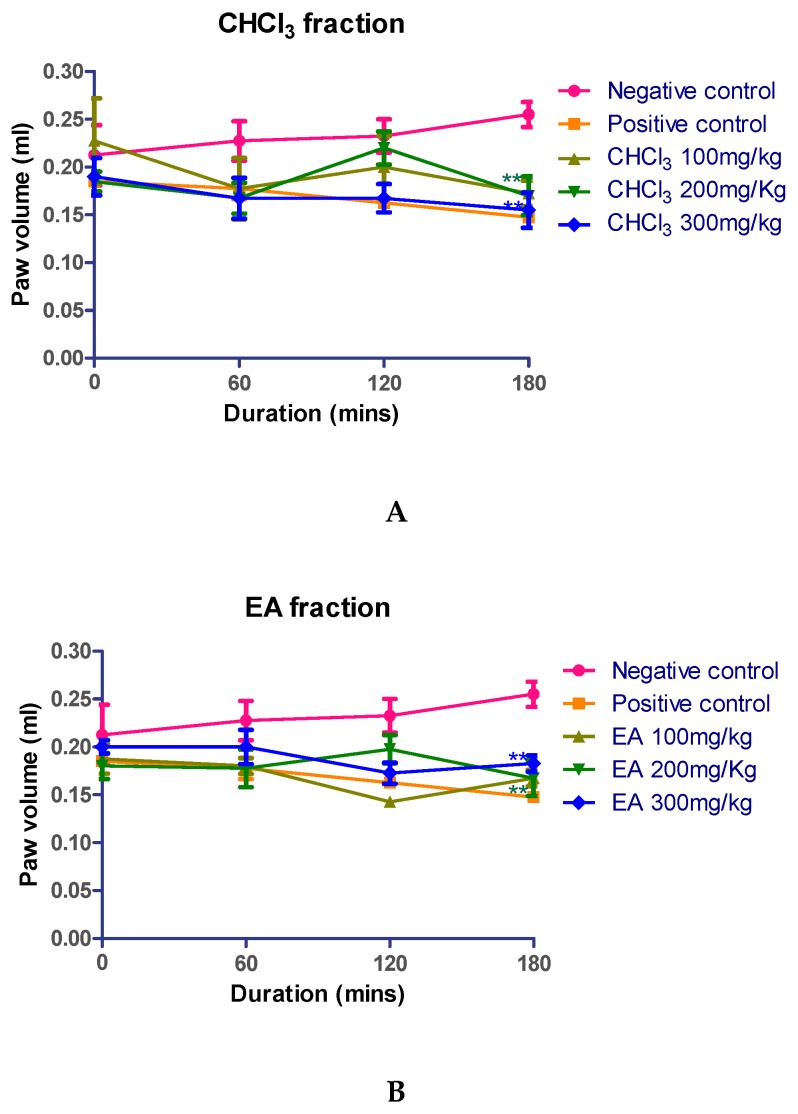
Anti-inflammatory activity of various fractions of *Kleinia pendula*. (**A**) Anti-inflammatory activity of various fractions of *Kleinia pendula* at different doses and time points. Chloroform (CHCl_3_) fraction at doses of 100, 200 and 300 mg/Kg body weight of the animal exhibited anti-inflammatory activity. (**B**) Ethyl acetate (EA) fraction at doses of 100, 200 and 300 mg/kg body weight of the animal exhibited anti-inflammatory activity. Diclofenac sodium (10 mg/kg) was used as a positive control, and standard saline was used as negative control. The values are represented as Mean ± SEM. Statistical analysis was done using one-way analysis of variance using Dunnett’s posthoc test.

The mice were administered with 100, 200, and 300 mg/kg fractions of *Kleinia pendula*. The volume of paw edema was recorded using a plethysmometer at 0, 60, 120, and 180 min after the drug administration. The chloroform and the ethyl acetate fractions at (200 and 300 mg/kg) of *Kleinia pendula* significantly reduced the paw edema at 180 min, and these results were comparable to the effect of the standard drug diclofenac sodium (10 mg/kg) used in this study (Figure 3).

### 2.4. Cytotoxic Assay

The cytotoxic profile of the methanolic extract and the fractions of *Kleinia pendula* were tested against breast cancer (MCF-7), liver cancer (HepG2), and colon cancer (HCT-116) cell lines. The methanolic extract, hexane and chloroform fractions of *Kleinia pendula* showed a more potent cytotoxic profile against all three cancer cell lines tested (Table 1). The IC_50_ values of the hexane and chloroform fractions of *Kleinia pendula* were comparable to the IC_50_ values of the standard drug doxorubicin. Whereas the ethyl acetate, butanol, and water fractions of *Kleinia pendula* showed a weaker cytotoxic profile against all three cancer cell lines tested. Importantly, the hexane fraction demonstrated a high cytotoxic effect against all three cancer cells tested, with IC_50_ values ranging from 0.07 µg to 0.19 µg.

### 2.5. Characterization of Metabolites from the Bioactive Fractions Using UPLC-PDA-ESI-MS

**Figure 4:**
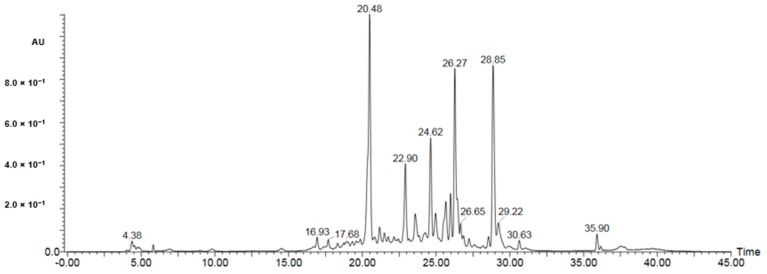
UPLC-PDA-ESI-MS in the bioactive ethyl acetate fractions.

**Figure 5:**
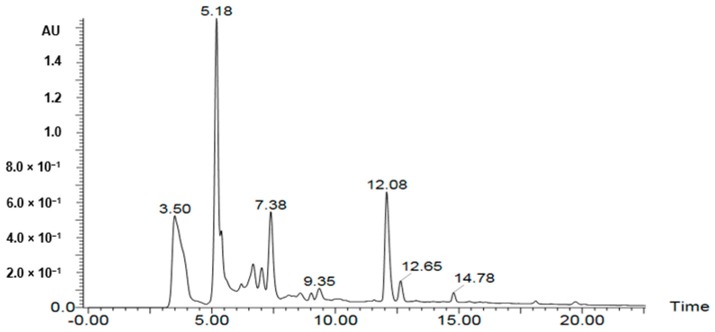
UPLC-PDA-ESI-MS in the chloroform fractions.

**Figure 6:**
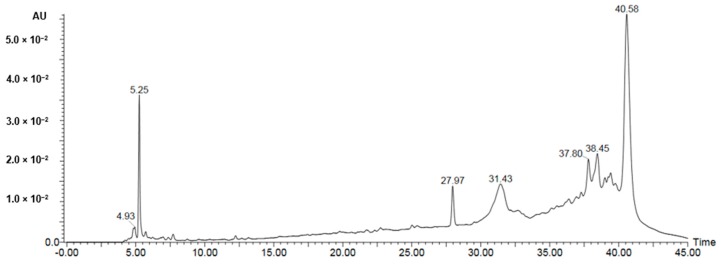
UPLC-PDA-ESI-MS in *n*-hexane fractions.

A UPLC-PDA-ESI-MS was used to characterize metabolites (Table 2, Table 3 and Table 4) in the bioactive ethyl acetate, chloroform, and *n*-hexane fractions (Figure 4, Figure 5 and Figure 6, respectively). Metabolite profiling revealed the presence of several compounds that belong to several classes of compounds to confirm the richness of *Kleinia pendula* with active metabolites. Polyol acids, phenolic acids, flavanoidal glycosides and tannins were found in the ethyl acetate and chloroform fractions. Hexane fraction revealed the presence of phenolic acids, diterpene glycosides, triterpenoids and fatty acids.

## 3. Discussion

This study demonstrates the in vivo analgesic and anti-inflammatory potential of the solvent fractions of *Kleinia pendula* for the first time. The hexane fraction of *Kleinia pendula* showed the most potent cytotoxic effect against three cancer cell lines (MCF7, HepG2 7 HCT-116).

The oral acute toxicity studies with fractions of *Kleinia pendula* at the dose of 2000 mg/kg did not produce any mortality, and no physical or behavioral changes were observed in the experimental animals during the study period. These results indicated that the estimated LD_50_ value of all the tested fractions was above 2000 mg/kg.

The aversive effect of the thermal stimulus from Eddy’s hot plate test was demonstrated by the subject mice licking their paws. This hot plate method demonstrates central nociceptive reactivity, rather than a simple reflex reaction [38]. In this study, the average latency period was increased when the ethyl acetate, chloroform, and the *n*-hexane fractions of *Kleinia pendula* were administered. The results demonstrated the analgesic effect of the various fractions of *Kleinia pendula*, probably due to the central mechanisms. Medicinal plants used as analgesics such as *Tamarindus indica* and *Curucuma zedoaria* have been shown to increase the average latency period [39,40].

In the study for anti-inflammatory activity, acute inflammation was induced in the sub-plantar region using carrageenan [41]. The initial phase of the inflammation (approximately 0–1.5 h) is ascribed to the release of histamine and serotonin. The second phase (1.5–2.5 h) is mainly mediated by bradykinin and the third phase (beyond 2.5 h) by the overproduction of the prostaglandins, leukotrienes [38]. The results showed that the chloroform fraction at a dose of 100 mg/kg effectively inhibited inflammation. Thus, the anti-inflammatory activity of the chloroform fraction of *Kleinia pendula* could be coupled with the inhibition of the release and the biosynthesis of histamine, serotonin, and leukotrienes. These fractions may have an antagonistic potential towards histamine and serotonin receptors, reflecting the initial phase of inflammation. *Kleinia pendula* has been used in Saudi and Yemeni traditional medicine to treat inflammation of the ear (Otitis) [14]. The decoction of the fresh succulent *Kleinia pendula* has been used to treat swollen body parts in Ethiopian traditional medicine [13]. Recently, the in vitro anti-inflammatory potential of a member of the genus *Kleinia* (*Kleinia odora*) has been reported [42]. Moreover, the methanolic extract of *Kleinia pendula* is reported to contain flavonoids and terpenoids [3]. The bioactive ethyl acetate, chloroform fractions were subjected to UPLC-PDA-ESI-MS metabolite screening. The major metabolites identified in the ethyl acetate fractions include Dicaffeoyl quinic acid [21], feruloyl-qunic acid [15], chlorogenic acid [17], sinapic acid hexoside [20], trihydroxyphenethyl-*O*-rhamnopyranosyl-(1,6)-4-*O*-caffeoyl-glucopyranoside [19], isorhamnetin-3-*O*-glucoside-7-*O*-rhamnoside [18], apigenin-6,8-di-C-glucoside and chlorogenic acid. The major metabolites identified in the chloroform fraction include HHDP-galloylglucose [26], dihydroxy-4-methoxylisoflavan [27], trihydroxyphenethyl-*O*-rhamnopyranosyl-(1-6)-4-*O*-caffeoyl-glucopyranoside [19], Glycycoumarin hydroxylate glucuronide [22,23] and sulfate conjugate of glycycoumarin [22,23].

The observed anti-inflammatory activity could be attributed to the presence of caffeoyl-quinic acid derivatives [43], protocatechuic [44], and chlorogenic [45] acids in the chloroform and ethyl acetate fractions respectively. Additional studies with isolated compounds are needed to corroborate this.

Chronic inflammation may increase the risk for various cancers. This is supported by epidemiological evidence that shows anti-inflammatory agents can be used for the prevention and treatment of cancer [46]). In this study, the methanolic extract, hexane, and chloroform fractions of *Kleinia pendula* showed potent cytotoxicity against breast cancer (MCF-7), liver cancer (HepG2), and colon cancer (HCT-116) cell lines, although the mechanisms are currently unclear. Significantly, the hexane fraction showed the most potent cytotoxic effect against all the cancer cell lines tested. The UPLC-PDA-ESI-MS metabolite screening of bioactive hexane fraction revealed the presence of major metabolites including tormentic acid [34], 5-hydroxyadvisiosidec [36], citrostadienyl [37], dehydrated derivative of tinosposinenside B [30] and tinocrisposide [30]. The observed cytotoxic activity could be attributed to the presence of fatty acids and triterpenoid (tormentic acid [47]) in the hexane fraction. Additional studies with isolated compounds are needed to corroborate this. This is in contrast to the earlier report where the methanolic and aqueous extract of *Kleinia pendula* was reported to have no significant cytotoxic activity against human urinary bladder cancer cell lines (5637) and breast cancer cell lines (MCF-7) [3].

## 4. Materials and Methods

### 4.1. Plant Material

Fresh parts of *Kleinia pendula* were collected during January 2017 from the Faifa Mountains in the southwest region of Saudi Arabia. *Kleinia pendula* is an annual or perennial herb, pendulous, succulent, leafless, stem cylindrical, branched. Leaves reduced to subulate scales upto 1.5 cm long, these arising from wart-like outgrowths on stems. Individual stems are joined and brittle, 20–30 cm long and 1.5 to 2 cm in diameter. The plant material was identified by a botanist in the Biology Department, College of Science, King Khalid University, and prepared for extraction.

### 4.2. Preparation of Extract and Fractions

The fresh aerial parts of *K. pendula* (4 kg) were pulverized and extracted by maceration with methanol (4 × 3 L) at room temperature. The methanolic extract was filtered and evaporated at a temperature of 40 °C using a rotary evaporator. The dried methanolic extract (200 g, 5%) was subsequently re-dissolved in water (300 mL) and partitioned successively several times with n-hexane (3 × 300 mL), chloroform (3 × 300 mL), ethyl acetate (3 × 300 mL), and *n*-butanol (3 × 300 mL) to provide the corresponding fractions. The fractions were evaporated using a rotary evaporator under reduced pressure at 40 °C to yield fractions of *n*-hexane (14 g, 7%), chloroform (8 g, 4%), ethyl acetate (20 g, 10%) and *n*-butanol (35 g, 17.5%) and the remainder of the aqueous fraction (95 g, 17.5%). The dried fractions were stored at −20 °C until used for screening of analgesic, anti-inflammatory, and cytotoxic activities.

### 4.3. Animals

All experiments were performed on Swiss mice of both sexes weighing between 20 g to 25 g. The animals were maintained on a 12 h light/dark cycle, with water and food ad libitum until use. This study was carried out according to the Ethics Committee, King Khalid University, Abha, Kingdom of Saudi Arabia, and followed the recommendations of the National Institutes of Health Guide for Care and Use of Laboratory Animals (Publication No. 85-23, revised 1985).

### 4.4. Acute Toxicity Studies

Acute toxicity studies of the *n*-hexane, chloroform and ethyl acetate fractions of *Kleinia pendula* were carried out at a dose of 2000 mg/kg in female mice, as per OECD 420 guidelines. The animals were randomly divided into four groups (*n* = 8). A control group having normal saline solution (0.9%) 10 mL/kg by oral route was compared with single dose of 2000 mg/kg; p.o. of the three fractions. The animal behavior and mortality index were closely observed for the first 3 h then at an interval of 4 h during the next 48 h) [48].

### 4.5. Measurement of Analgesic Activity

The analgesic activity was assessed by using an Eddy hot plate method [38] on mice weighing 20–30 g. Mice were placed on the hot plate, which consisted of an electrically heated surface. The temperature of the hot plate was maintained at 55 °C. Responses such as jumping, withdrawal of the paws, and licking of the paws were observed. The time period (latency period) between the placement of the animals on the hot plate, and their responses was recorded. The experimental animals were separated into 5 groups of 8 animals in each group (Group 1: negative control; Group 2: positive control; Group 3: 100 mg/kg of respective fraction; Group 4: 200 mg/kg of respective fraction; Group 5: 300 mg/kg of respective fraction). The respective groups of animals were administered with 100, 200, and 300 mg/kg of chloroform, ethyl acetate, and *n*-hexane fractions of *Kleinia pendula* orally and the latency period was recorded at 0, 30, 60, and 120 min after drug administration. Diclofenac sodium 10 mg/kg was used as a standard drug, and the control group of animals were treated with standard saline.

### 4.6. Measurement of Anti-Inflammatory Activity

Paw edema was introduced by injection of 30 µl of 1% *w*/*v* carrageenan suspension on the plantar surface of a hind paw, and the edema was measured using a plethysmometer [49]. The experimental animals were separated into 5 groups of 8 animals in each group (Group 1: negative control; Group 2: positive control; Group 3: 100 mg/kg of respective fraction; Group 4: 200 mg/kg of respective fraction; Group 5: 300 mg/kg of respective fraction. The respective groups of animals were administered with 100, 200, and 300 mg/kg of chloroform and ethyl acetate fractions of *Kleinia pendula* orally, and the volume of the paw edema was measured at 0, 60, 120, and 180 min after the drug administration. Diclofenac sodium 10 mg/kg was used as a standard drug, and the control group of animals was treated with standard saline.

### 4.7. Cell Culture

Human hepatocellular carcinoma cell line (HepG-2), colorectal adenocarcinoma cell line (HCT-116), and breast adenocarcinoma cell line (MCF-7) cells were obtained from the American Type Culture Collection (ATCC). All the cell lines were cultured in RPMI-1640 medium containing penicillin and streptomycin (100 g/mL; 100 units/mL) and heat-inactivated fetal bovine serum (10% *v*/*v*) in a humidified, 5% (*v*/*v*) CO_2_ atmosphere at 37 °C) [48].

### 4.8. Cytotoxicity Studies

The cytotoxicity of various extract/fractions of *Kleinia pendula* was tested against human tumor cells using Sulphorhodamine B assay (SRB). Healthy growing cells were cultured in a 96-well tissue culture plate (3000 cells/well) for 24 h to allow attachment of the cells to the plate. Cells were exposed to the five different concentrations of each extract/fraction (0.01, 0.1, 1, 10, and 100 μg/mL); untreated cells (control) were added. Triplicate wells were incubated with the different concentrations for 72 h. and subsequently fixed with trichloroacetic acid (10% *w*/*v*) for 1 h at 4 °C. After several washing episodes, cells were stained with a 0.4% (*w*/*v*) SRB solution for 10 min in a dark place. Excess stain was washed with 1% (*v*/*v*) glacial acetic acid. After drying overnight, the SRB-stained cells were dissolved with tris-HCl and the color intensity was measured in a microplate reader at 540 nm. Doxorubicin was used a positive control. The linear relationship between the viability percentage of each tumor cell line and the extracts’ concentrations were analyzed to obtain the IC_50_ (dose of the drug which reduces survival to 50%) using SigmaPlot 12.0 software [50].

### 4.9. UPLC-PDA-ESI-MS Analysis of the Active Fractions

Mass spectrometric analysis (UPLC-PDA-ESI-MS) was performed using Waters ACQUITY Xevo Triple quadrupole (TQD) system, which consisted of an ACQUITY UPLC H-Class system and Xevo^TM^ TQD triple-quadrupole tandem mass spectrometer with an electrospray ionization (ESI) interface (Waters Corp., Milford, MA, USA). Acquity BEH C18 100 mm × 2.1 mm column (particle size, 1.7 µm) was used to separate analytes (Waters, Ireland). For the chloroform and hexane fractions, the solvent system consisted of 0.1% formic acid in water (A) and 0.1% formic acid in acetonitrile (B) by applying the following mobile phase gradient: 0–4 min, 30% B; 4–8 min, 35% B; 8–25 min, 70% B; 25–30 min, 30% B. For the ethyl acetate fraction, the solvent system consisted of 0.1% formic acid in water (A) and 0.1% formic acid in acetonitrile (B) with the following mobile phase gradient: 0–4 min, 15% B; 4–8 min, 20% B; 8–30 min, 55% B; 30–35 min, 90% B; 35–40 min, 15% B. The flow rate was 200 μL/min and the injection volume was 10 µL. The samples were dissolved in methanol at a concentration of 1 mg/mL then filtered through a filter of pore size 0.2 µm. The eluted compounds were detected at mass ranges from 100 to 1000 *m*/*z*. The MS scan was carried out at the following conditions: capillary voltage, 3.5 kV; detection at cone voltages, (20–95 V); radio frequency (RF) lens voltage, 2.5 V; source temperature, 150 °C; de-solvation gas temperature, 500 °C. Nitrogen was used as de-solvation and cone gas at a flow rate of 1000 and 20 L/h, respectively. System operation and data acquisition were controlled using Mass Lynx 4.1 software (Waters).

### 4.10. Statistical Methods

GraphPad Prism software was used for all animal experiments. The values are represented as mean ± SEM. Statistical analysis for significance (*p* < 0.05) was done using one-way analysis of variance using the Dunnetts posthoc test. SigmaPlot 12.0 software was used for the cytotoxicity assay.

## 5. Conclusions

In conclusion, the present study results show that the n-hexane and chloroform fractions of *Kleinia pendula* showed significant cytotoxic activity against breast, liver, and colon cancer cell lines, probably due to the fatty acids and terpenoid (tormentic acid). The ethyl acetate and chloroform fractions of *Kleinia pendula* showed a significant analgesic and anti-inflammatory activities due to phenolic acid content mainly caffeoylquinic acid derivatives, protocatechuic and chlorogenic acids). Therefore, these fractions of *Kleinia pendula* may be a novel source for the development of new plant-based analgesic, anti-inflammatory, and anticancer drugs.

## Figures and Tables

**Table 1:** Cytotoxic activities of extract/fractions of *Kleinia pendula* against different cancer cell lines.

Fractions/Extract	IC_50_ (μg/mL)		
	MCF-7 (µg)	HepG2 (µg)	HCT-116 (µg)
Methanol extract	3.2 ± 0.6	3.17 ± 0.69	4.8 ± 0.6
Hexane fraction	0.07 ± 0.03	0.19 ± 0.02	0.11 ± 0.01
Chloroform fraction	0.13 ± 0.07	0.24 ± 0.03	0.19 ± 0.15
Ethyl acetate fraction	90.22 ± 18.6	≥100	28.1 ± 5.3
Butanol fraction	≥100	≥100	≥100
Water fraction	≥100	≥100	≥100
Doxorubicin	0.014 ± 0.008	0.0065 ± 0.005	0.013 ± 0.0005

*p* values less than 0.05 are considered statistically significant. IC_50_ values of the fractions were significantly higher than that of positive control (Doxorubicin).

**Table 2:** Peak assignment using UPLC-PDA-ESI-MS of metabolites detected in ethyl acetate fraction (negative mode).

Peak	Retention Time	Identified Compd.	UV-Vis (λ Max)	[M − H]^−^ (*m*/*z*)	Fragment Ions (*m*/*z*)	Percentage (%)	Ref.
*Kp1*	4.2	Quinic acid	323	191	178, 173, 148, 110	0.8	[15].
*Kp2*	6.2	Protocatechuic acid	320	153	113, 105	0.5	[16].
*Kp3*	20.48	Di-caffeoyl quinic acid	246, 310	515	191	5.7	[15,17]
*Kp4*	21.32	Chlorogenic acid	246, 310	353	191	1.21	[17]
*Kp5*	21.98	Feruloyl-quinic acid	247,310	367	191	0.62	[15]
*Kp6*	22.9	Isorhamnetin-3-*O*-glucoside-7-*O*-rhamnoside	230, 262	623	315, 153	3.2	[18]
*Kp7*	23.09	Feruloyl-quinic acid	247,310	367	191	1.32	[15]
*Kp8*	24.6	Trihydroxyphenethyl-*O*-rhamnopyranosyl-(1-6)-4-*O*-caffeoyl-glucopyranoside	232, 282	621	487, 469	3.4	[19]
*Kp9*	25.72	Sinapic acid hexoside	310	385	223	4.82	[20]
*Kp10*	25.85	Di-caffeoyl-hexuronide derivative	328	710	355, 135, 113	2.5	[21]
*Kp11*	26.27	Chlorogenic acid	246, 310	353	191	4.95	[17]
*Kp12*	28.86	Feruloyl-quinic acid	333	367	191	5.1	[15]
*Kp13*	29.22	Sulphate conjugate of dimethyl gallic acid	260	277	197,163	1.6	[22,23]
*Kp14*	30.62	Di-methylgallic acid derivative	267	291	155	0.7	[22,23]
*Kp15*	35.9	Coumaroyl-shikimic acid	225	319	155	0.92	[15]
*Kp16*	38.9	Apigenin-6,8-di-C-glucoside	246, 310	593	297,135	1.6	[22,23]
*Kp17*	40.21	Formylipolamiidic acid	Undetected	419	401, 257, 155	0.9	[22,23]
*Kp18*	42.82	Procyanidin B3	330	579	453, 127	0.5	[24,25]

**Table 3:** Peak assignment using UPLC-PDA-ESI-MS of metabolites detected in the chloroform fraction (negative mode).

Peak	Retention Time	Identified Compd.	UV-Vis (λ Max)	[M − H]^−^ (*m*/*z*)	Fragment Ions (*m*/*z*)	Percentage (%)	Ref.
*Kp19*	3.5	Hexahydroxydiphenoyl (HHDP)-galloylglucose	274	633	301, 257, 229	5.4	[26]
*Kp20*	5.18	Dihydroxy-4-methoxyl isoflavan	226, 284	271	227, 135	3.75	[27]
*Kp21*	5.2	Gallocatechin	274	305	179	1.29	[17]
*Kp22*	6.2	HHDP-galloylglucose	274	633	301, 257, 229	1.3	[26]
*Kp23*	6.9	Trihydroxyphenethyl-*O*-rhamnopyranosyl-(1-6)-4-*O*-caffeoyl-glucopyranoside	232, 282	621	487, 469	3.4	[19]
*Kp24*	7.38	unidentified	230, 290	604	582, 462, 342	2.38	[28]
*Kp25*	9.2	Methylretusin	230, 283	297	281, 239	0.18	[27]
*Kp26*	9.35	Acyl-feruloyl-4-*O*-caffeoyl-quinic acid	220, 232	571	277, 191	0.95	[15]
*Kp27*	12.08	Glycycoumarin hydroxylate glucuronide	258	559	338	3.29	[22,23]
*Kp28*	12.65	Sulfate conjugate of glycycoumarin	283	447	367	2.01	[22,23]
*Kp29*	14.78	trisgalloyl (hexahydroxydiphenoyl) glucose derivative	275	907	765,191	0.72	[29]

**Table 4:** Peak assignment using UPLC-PDA-ESI-MS of metabolites detected in hexane fraction (negative mode).

Peak	Retention Time	Identified Compd.	[M − H]^−^ (*m*/*z*)	Fragment Ions (*m*/*z*)	Percentage (%)	Ref.
*Kp30*	4.2	Tinosposinenside B	581	379, 343, 297	0.21	[30]
*Kp31*	4.93	Amritoside A	555	537, 513	0.42	[30]
*Kp32*	4.95	Isocryptotanshinone II	297	225, 211	0.8	[31]
*Kp33*	5.23	Linolenic acid	277	250, 219	3.5	[31]
*Kp34*	11.3	Octadecadienoic acid derivative	265	249, 179	0.2	[31]
*Kp35*	14.2	Tanshinone IIB	311	275, 250	0.73	[32]
*Kp36*	25.6	Salvianolic acid G	339	277, 249	0.35	[33]
*Kp37*	27.97	Tinocrisposide	535	521, 355	1.9	[30]
*Kp38*	31.43	Tormentic acid	487	469	4.9	[34]
*Kp39*	34.3	6,7-Dehydroroyleanone	315	297, 216	1.87	[35]
*Kp40*	36.8	Salvianolic acid D	417	197, 175, 135	1.2	[31]
*Kp41*	37.80	Dehydrated derivative of Tinosposinenside B	419	297	3.75	[30]
*Kp42*	38.45	5-Hydroxydavisiosidec	513	197	4.89	[36]
*Kp43*	39.5	Citrostadienyl	432	419	4.3	[37]
*Kp44*	40.58	Unidentified	319	305, 291, 277	6.75	n.d.
